# Lnc-MCEI mediated the chemosensitivity of esophageal squamous cell carcinoma via miR-6759-5p to competitively regulate IGF2

**DOI:** 10.7150/ijbs.47051

**Published:** 2020-09-16

**Authors:** Guangming Liu, Wei Guo, Guang Chen, Wencan Li, Youbin Cui, Junjie Qin, Jing Peng

**Affiliations:** 1 Department of Gastroenterology, The First Hospital of Jilin University, Changchun 130021, P.R. China.; 2 Department of Hemotology, The First Hospital of Jilin University, Changchun 130021, P.R. China.; 3 Department of Cardiothoracic Surgery, The Affiliated Zhuzhou Hospital of Xiangya Medical College CSU, Zhuzhou 412000, China.; 4 Department of Thoracic Surgery, The First Hospital of Jilin University, Changchun 130021, P.R. China.

**Keywords:** lnc-MCEI, miR-6759-5p, esophageal squamous cell carcinoma, chemosensitivity, IGF2

## Abstract

Large amounts of long non-coding RNAs (lncRNAs) have been annotated whereas most of them have not been functionally characterized. Here we identified lncRNA ENST00000441932 as an oncogenic lncRNA in esophageal squamous cell carcinoma (ESCC) and named lnc-MCEI (lncRNA mediated the chemosensitivity of ESCC by regulating IGF2). What's more, the effect of lnc-MCEI on the chemosensitivity of ESCC was further evaluated. Bioinformatics analysis demonstrated that lnc-MCEI was involved in the tumorigenesis of ESCC and lnc-MCEI levels were significantly increased in ESCC cells and tissues. Additionally, lnc-MCEI knockdown retarded cell proliferation, colony formation of ESCC cells, but induced cell apoptosis. Moreover, lnc-MCEI knockdown significantly improved the chemosensitivity of ESCC to cisplatin (DDP) both *in vivo* and *in vitro*. Further mechanisms disclosed that lnc-MCEI functioned as a competing endogenous RNA (ceRNA) via sponging miR-6759-5p and IGF2 was a target of miR-6759-5p. Meanwhile, we found that IGF2 suppressed chemosensitivity of ESCC cells via PI3K/AKT pathway. These data suggested that lnc-MCEI was an oncogenic lncRNA and lnc-MCEI knockdown enhanced chemosensitivity of ESCC cells to cisplatin by targeting miR-6759-5p /IGF2/PI3K/AKT axis.

## Introduction

Esophageal cancer ranks eighth among all tumors and sixth among causes of cancer deaths, with at least 456,000 new cases annually worldwide 1. The morbidity of esophageal cancer is more than three times higher in men than in women because alcohol abuse and smoking are considered two of the most common risk factors for esophageal cancer and it is more common in less economically developed rural areas 2. Esophageal carcinoma consists of esophageal squamous cell carcinoma (ESCC) and esophageal adenocarcinoma (EAC) 3. Compared with EAC, ESCC is the dominant histological type, accounting for up to 80% of all esophageal cancer cases 4. Cisplatin (DDP) and 5-Fluorouracil (5-FU) have been regarded as the first-line drugs for the ESCC treatment 5. Due to the chemoresistance, only 15-25 % of patients survive beyond 5 years despite the combination of surgery with adjuvant therapies such as chemotherapy utilized to treat ESCC 6. Therefore, it is extremely vital to explore the underlying molecular mechanisms of chemoresistance and develop new strategies to increase the efficacy of chemotherapy drugs.

Long non-coding RNAs (lncRNAs) are considered to be non-coding RNAs with >200 nucleotides (nt) in length and have no protein coding abilities 7. LncRNAs account for at least 80 % of the human genome and have diversity of biological functions 8. Emerging studies have demonstrated that lncRNAs are involved in tumorigenesis of various tumors such as gastric cancer 9, breast cancer 10, osteosarcoma 11, colorectal cancer 11, glioma 12, and so on. Additionally, lncRNAs have been proved to be implicated in the tumorigenesis of esophageal squamous cell carcinoma. Previous studies demonstrated that lncRNA CASC9 promotes ESCC progression with upregulated LACM2 by interaction with CREB-binding protein in the nucleus 13. Another study indicated that lncRNA CASC9 negatively regulate PDCD4 by binding with enhancer of zeste homolog 2 (EZH2) 14. In addition, studies showed that lncRNA DUXAP10 promotes tumorigenesis of ESCC by epigenetically silencing p21 15. LncRNA GHET1 facilitates the proliferation and invasion of ESCC cells 16. However, there are still plenty of lncRNAs involved in the ESCC have not been identified. In the present study, the role of lnc-MCEI in the ESCC progression and chemoresistance was evaluated.

Thus, our study was aimed to explore the role of lnc-MCEI in the ESCC progression and chemoresistance. Bioinformatics analysis demonstrated that lnc-MCEI might participate in ESCC chemosensitivity of ESCC to cisplatin and further experiments validates this both *in vivo* and *in vitro*. Therefore, this study revealed that lnc-MCEI promotes ESCC tumorigenesis and chemoresistance and was a potential therapeutic target for ESCC.

## Materials and Methods

### Tissue sample collection

A cohort of tumor and adjacent normal tissues were collected from 50 patients diagnosed with ESCC in the First Hospital of Jilin University from August, 2013 to August, 2018 under the approval of Institutional Review Board and Human Ethics Committee of the hospital. Patients were performed complete surgical resection (esophagectomy) prior to adjuvant chemo- or radiotherapy and ESCC samples were collected. Adjacent normal tissues were taken from the side of the tumor at least 5 cm away from the edge of the tumor. All tissues were stored at -80 ℃ until use and all the patients have signed the informed consent form.

### Bioinformatics analysis

R package *limma* was used to obtain the differential expressed genes (DEGs) from the microarray data (GSE89102) which was available in the Gene Expression Omnibus (GEO) repository 17. 3225 DEGs were finally identified (BH-corrected *p* value < 0.01). Probe information has been reported previously 17, 18. Microarray data (GSE104958) was further used to analyze the expression of long non-coding RNA (lncRNA)-MCEI in tissues with pathological complete response (pCR) compared to non-pathological complete response (non-pCR).

### Cell culture, plasmids construction and transient transfection

Human esophageal epithelial cell line (Het-1A) and ESCC cell lines (TE-1, KYSE-30, EC109 and EC9706) were used in the present study. Het-1A cell line was obtained from the American Type Culture Collection (Manassas, VA, United States) and TE-1 cell line was purchased from Chinese Academy of Medical Sciences (Beijing, China). In addition, KYSE-30 and EC109 cell lines were derived from CoBioer (Nanjing, China) and EC9706 cell line was gotten from Shanghai Huiying (Shanghai, China). KYSE-30 or EC109 cells were transfected with the small interfering RNA (siRNA) targeting lnc-MCEI (si-MCEI), miR-6759-5p mimics, miR-6759-5p inhibitor at 50 % confluence. Lnc-MCEI and negative control (NC) hairpin RNA (shRNA) sequences were purchased from GenePharma (Shanghai, China) and cloned into the pBS/U6 vector (Addgene) for lnc-MCEI knockdown. Additionally, the IGF2 expression plasmids construction and transient transfection were performed as the protocol previously described 19.

### Quantitative real-time polymerase chain reaction (qPCR)

The mRNA levels of target genes were examined by qPCR as the protocol previously described 20. For mRNA and miRNA, GAPDH and U6 were used as internal controls. Primer pairs of lnc-MCEI, U6 snRNA and GAPDH were purchased from GenePharma (Shanghai, China) and primers of IGF2 used for amplification in this study are:

- 5′-CGCCGAACCAAAGTGGATTA-3′ (forward) and;
- 5′-GGAGAGACAGAGTGAACGTGA-3′ (reverse).

### MTT, colony formation, migration, invasion assays and flow cytometry

Cell proliferation were analyzed by MTT assays and cells colonies subjected to different treatments were stained and counted 10-14 days after seeding as previously described 21. Transwell chambers were used to evaluate the migration and invasion capacities of cancer cells and assays were done as previously described 21. The percentage of apoptotic cells were determined by an Annexin V-FITC/PI Apoptosis Detection Kit (Sigma-Aldrich). All the experimental sets were performed in triplicate.

### Dual luciferase reporter assays

Wild-type (WT) or mutant (MUT) 3'-UTR of lnc-MCEI and IGF2 were cloned into the firefly-tagged pGL3 promoter luciferase vector (Promega, Madison, WI, USA) and then were co-transfected into HEK-293 cells with the Renilla control luciferase vector (Promega). Additionally, cells were co-transfected with miR-6759-5p mimics or miR-6759-5p inhibitor for another 48 hours. Luciferase activities were detected with a dual luciferase assay system (Promega) and results were presented as the ratio of firefly/Renilla.

### Western blotting

Total protein extraction and western blotting was performed as previously described 22. Primary IGF2 antibody (ab18954; 1:1000) and GAPDH antibody (ab181602; 1:2000) were purchased from abcam (MA, USA), and p-AKT (BS4009, 1:1000) and t-AKT (BS1810; 1:1000) were obtained from Bioworld Technology (MN, USA).

### RNA immunoprecipitation (RIP) assays

For RIP assays, a Magna RIP™ RNA-Binding Protein Immunoprecipitation Kit (Millipore, USA) was utilized according to the manufacturer's protocols. Then the lnc-MCEI mRNA levels were determined by qRT-PCR.

### Immunohistochemistry (IHC)

Immunostaining of Ki67 were carried out as previously described 23. Briefly, sections were incubated with Ki67 antibody (1:200, Sigma-Aldrich, St. Louis, MO, USA) at 4 °C overnight. Horseradish peroxidase (HRP)-conjugated secondary antibodies were further incubated and visualized with 3, 3'-diaminobenzidine (DAB).

### Xenograft mouse model

Twenty-four male BALB/c nude mice (six-week-old and six mice in per group) were randomly divided into four groups including NC, sh-lnc-MCEI, DDP and sh-lnc-MCEI+DDP. 3×10^6^ EC109 cells of sh-control or MCEI-shRNA in 100 μL PBS were administered via subcutaneous injection into the right groin of the nude mice. The mice were intraperitoneally injected of DDP (1 mg/kg) from the second week every 3 days. The formula (volume (mm^3^) = (length × width^2^)/2) was used for the calculation of tumor volume every 3 days. Mice were sacrificed on day 18 post injection and tumors were harvested and weighed 24. Experimental procedures were approved by the Institutional Animal Use and Care Committee of the First Hospital of Jilin University.

### Statistical analysis

Results (mean ± SD) were analyzed with a one-way ANOVA or Student's t-test (SPSS 19.0, Chicago, IL, USA). The correlation between miR-6759-5p and lnc-MCEI in ESCC tissues was analyzed by using Spearman correlation analysis. *P* < 0.05 was considered to be statistically significant.

## Results

### Up-regulated lnc-MCEI was implicated in the promotion of ESCC progression and chemoresistance

**Figure 1:**
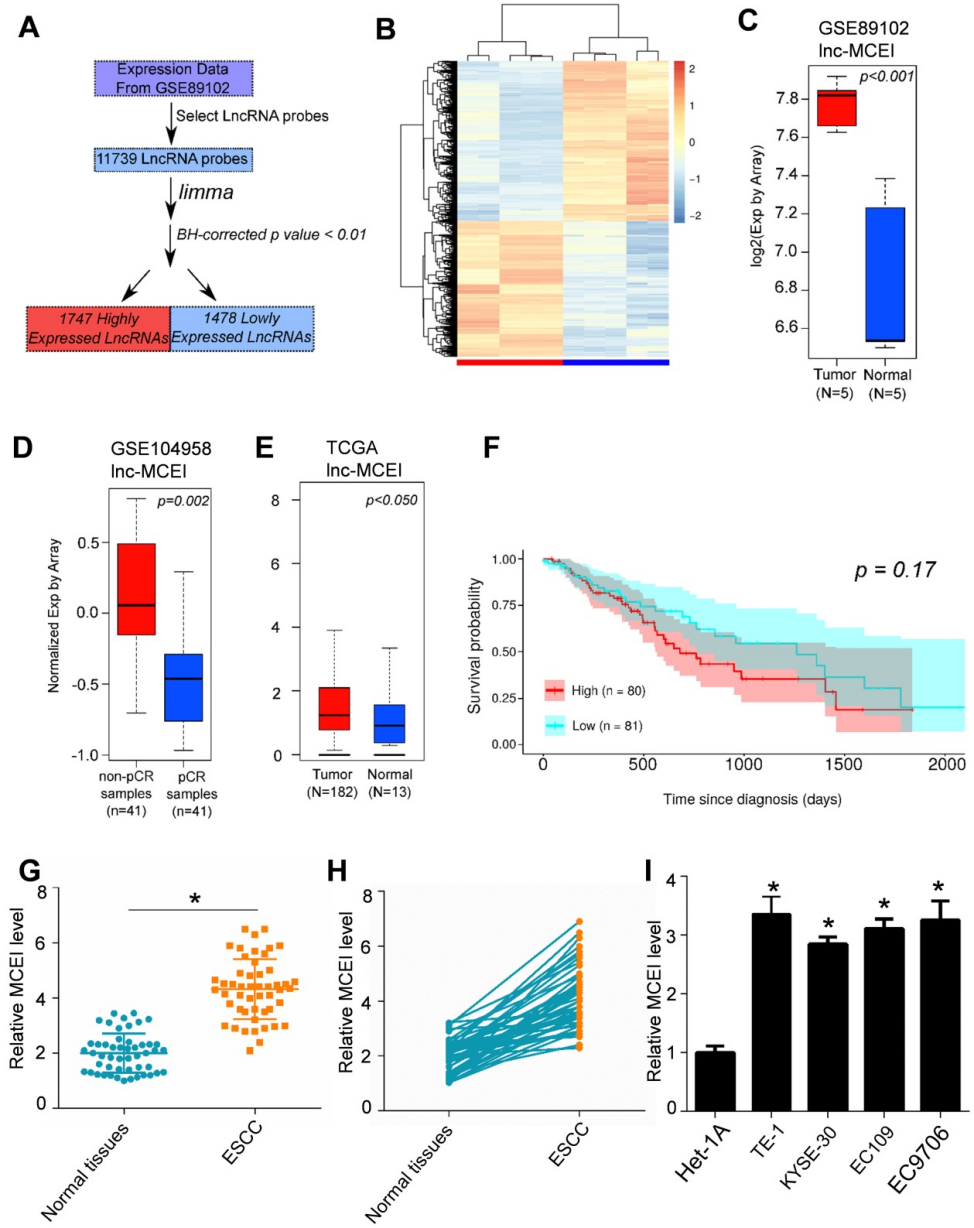
** Lnc-MCEI was up-regulated in the ESCC tissues and tissues with non-pathological complete response (non-pCR). A.** Flow diagram of data analysis based on the published microarray data (GSE89102) with R package *limma*. BH-corrected *p* value < 0.01 was considered to be statistically significant. **B.** A heat map demonstrated the significantly differential expressed genes (DEGs). The bottom bar denotes the sample types that the red indicates tumor tissues and the blue indicates normal tissues. **C.** Lnc-MCEI was one of most significantly up-regulated genes in ESCC tissues among the DEGs. **D.** Significantly up-regulated lnc-MCEI in tissues with non-pathological complete response (non-pCR) compared to pathological complete response (pCR). **E.** The significantly higher lnc-MCEI expression in tumor tissues compared with the normal tissues according to data from The Cancer Genome Atlas (TCGA) RNA-Seq database. **F.** Increased expression of lnc-MCEI was significantly associated with poor patient survival (*P* = 0.17). **G.** The significantly up-regulated lnc-MCEI in ESCC tissues were observed compared to the normal controls. N = 50. **H.** Lnc-MCEI expression was significantly higher in ESCC tissues than the matched normal tissues. N = 50. **I.** Lnc-MCEI was significantly up-regulated in ESCC cell lines compared to the human esophageal epithelial cell line (Het-1A). *P < 0.05.

To determined candidate genes involved in esophageal squamous cell carcinoma (ESCC) chemoresistance, a bioinformatics analysis was carried out based on the published microarray data (GSE89102) obtained from the Gene Expression Omnibus (GEO). Differential expressed genes (DEGs) were then examined between the 5 paired ESCC tissues and normal tissues by using the microarray information and 1747 significantly up-regulated and 1478 significantly down-regulated DEGs were finally identified (*P* < 0.01; **Figure 1A and 1B**). Further analysis was performed and we found that lnc-MCEI was one of most significantly up-regulated genes in ESCC tissues (**Figure 1C**). Moreover, the data series (GSE104958) was utilized and lnc-MCEI was also significantly increased in ESCC tissues with non-pathological complete response (non-pCR) compared to the tissues with pathological complete response (pCR) after chemotherapy (**Figure 1D**). Additionally, data from The Cancer Genome Atlas (TCGA) RNA-Seq database was analyzed to support this and the lnc-MCEI levels in tumors were significantly higher than normal tissues (**Figure 1E**). Next, the association of lnc-MCEI expression with patient survival was further evaluated according to the TCGA dataset. As shown in **Figure 1F**, increased expression of lnc-MCEI was significantly associated with poor patient survival (*P* = 0.17). Based on the above bioinformatics results, the increased lnc-MCEI expression in ESCC tissues compared to the normal controls or matched normal tissues was confirmed (N = 50; **Figure 1G and 1H**). In addition, up-regulated lnc-MCEI expression in ESCC cell lines compared to the human esophageal epithelial cell line (Het-1A) was also observed (**Figure 1I**). These results demonstrated that up-regulated lnc-MCEI was implicated in the promotion of ESCC progression and chemoresistance.

### Lnc-MCEI knockdown significantly inhibits the malignant biological behaviors of ESCC cells

**Figure 2:**
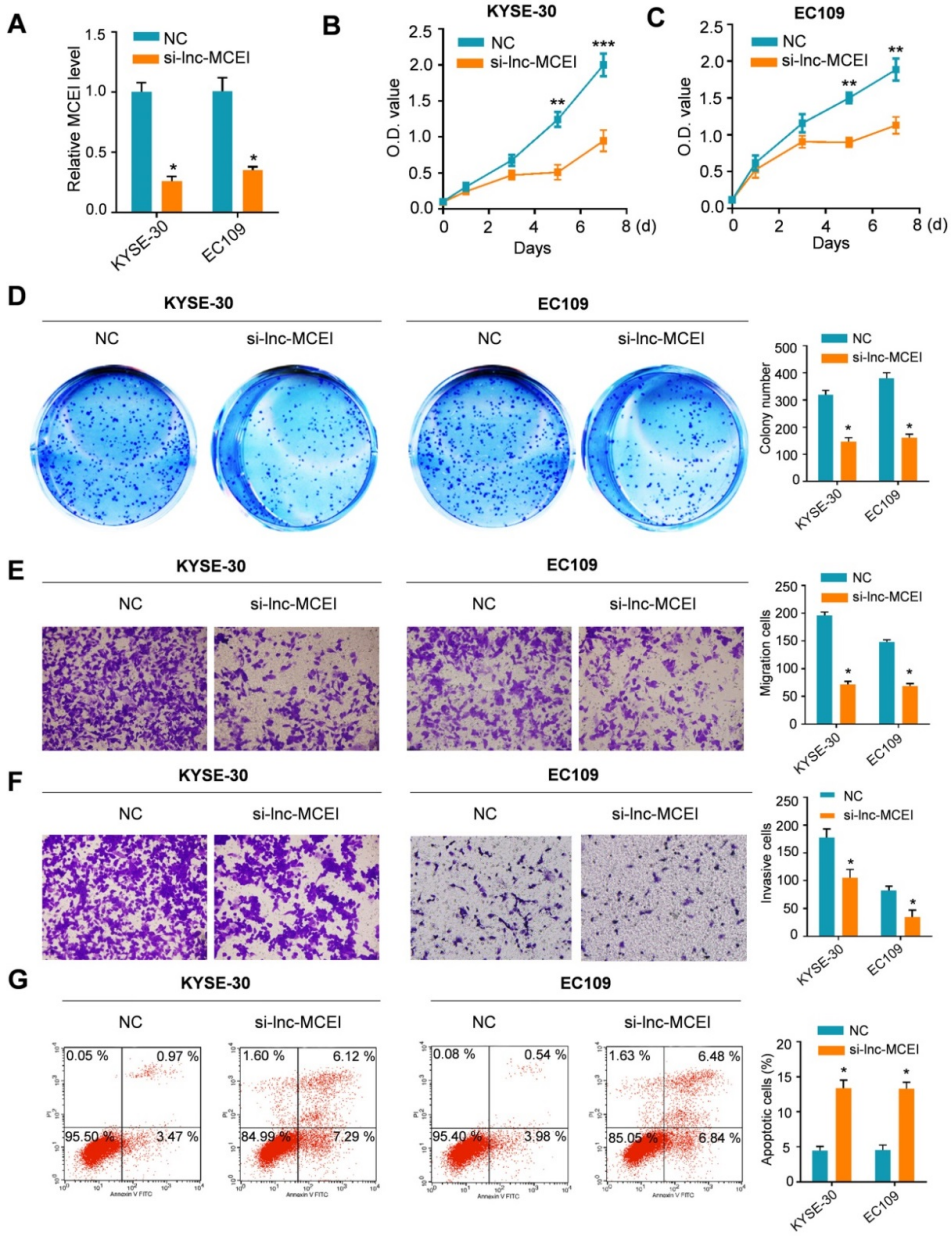
** Lnc-MCEI knockdown significantly inhibits the proliferation, migration and invasion of ESCC cells. A.** Lnc-MCEI knockdown was performed in both KYSE-30 and EC109 cells and the knockdown efficiency were detected by qRT-PCR. **B.** MTT assays in KYSE-30 cells. **C.** MTT assays in EC109 cells. **D.** Colony formation assays in both KYSE-30 and EC109 cells. **E.** Migration and invasion assays in both KYSE-30 and EC109 cells. **F.** Invasion assays in both KYSE-30 and EC109 cells. **G.** The results of flow cytometry indicated that lnc-MCEI knockdown significantly increased the percentage of apoptotic cells in both KYSE-30 and EC109 cells. *P < 0.05, **P < 0.01 and ***P < 0.001.

Subsequently, the role of lnc-MCEI in tumorigenesis was evaluated by knockdown strategies in KYSE-30 and EC109 cells. As shown in **Figure 2A,** the lnc-MCEI expression was examined to confirm the knockdown efficiency in both KYSE-30 and EC109 cells. Then the MTT and colony formation assays were used to examine the effect of lnc-MCEI on cell growth. The results suggested that cell proliferation and clone formation of KYSE-30 and EC109 cells with lnc-MCEI knockdown were significantly suppressed compared to the negative controls (NC;** Figure 2B-D**). Accordingly, cells with lower lnc-MCEI levels exhibited significantly inhibited migration and invasion properties (**Figure 2E and 2F**). Furthermore, lnc-MCEI knockdown significantly increased the percentage of apoptotic cells in both of the cell lines (**Figure 2G**). These data suggested that lnc-MCEI knockdown significantly inhibited the malignant biological behaviors of ESCC cells.

### Lnc-MCEI knockdown sensitizes ESCC cells to DDP

**Figure 3:**
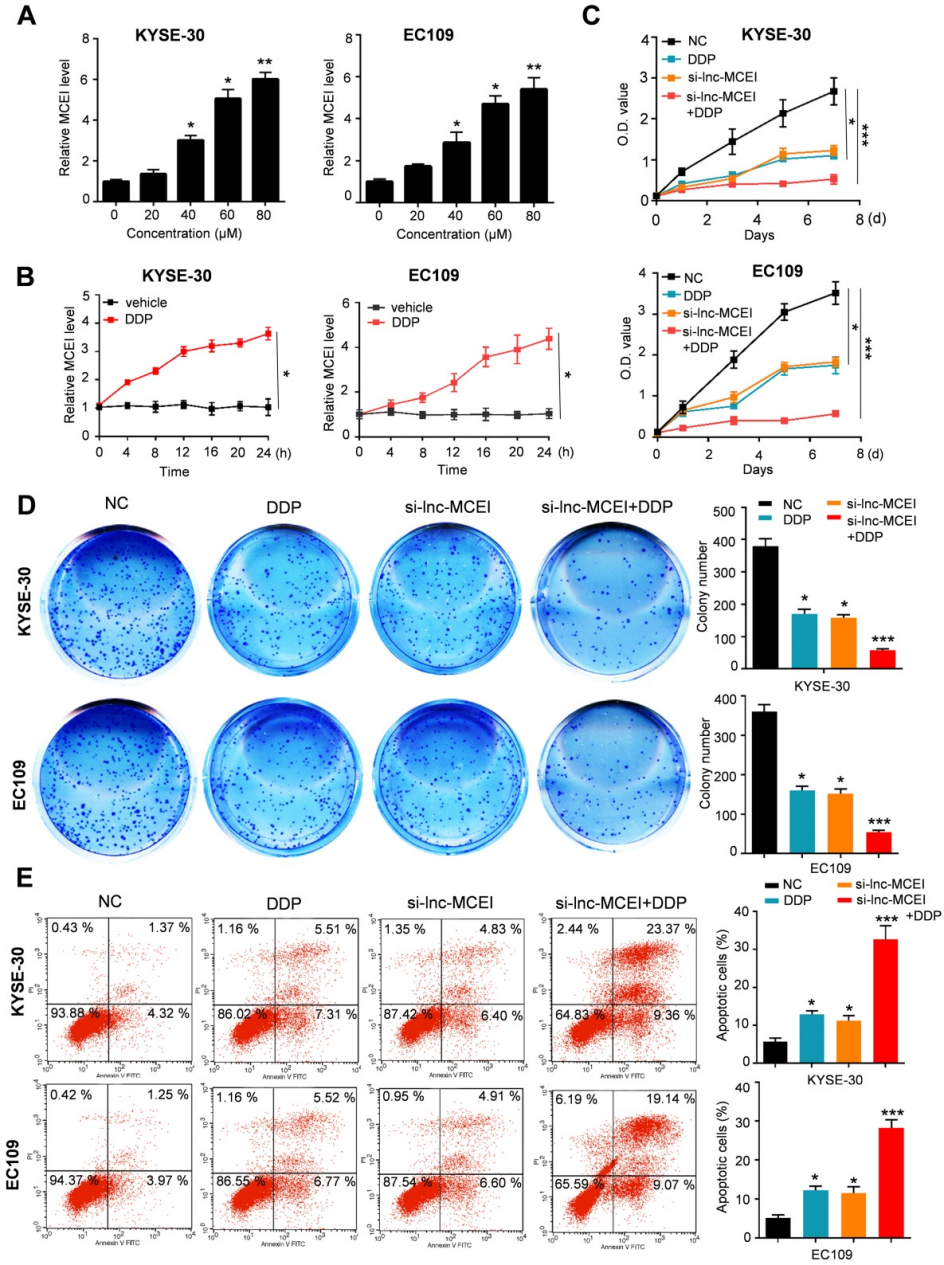
** Lnc-MCEI knockdown sensitizes ESCC cells to DDP. A** and **B** Lnc-MCEI expression significantly increased in a time- and dose-dependent manner in KYSE-30 and EC109 cells subjected to DDP. **C.** Both KYSE-30 and EC109 cells were treated with si-lnc-MCEI alone or combined with DPP and MTT assays were performed to show the cell proliferation with various treatments. **D.** Colony formation assays in various groups. **E.** The percentage of apoptosis in various groups were tested by flow cytometry. *P < 0.05, **P < 0.01 and ***P < 0.001.

Next, to determine the effect of lnc-MCEI on chemosensitivity, KYSE-30 and EC109 cells were treated with cisplatin (DDP) combined with lnc-MCEI knockdown or not. As shown in **Figure 3A and 3B**, lnc-MCEI expression significantly increased in a time- and dose-dependent manner in KYSE-30 and EC109 cells subjected to DDP. More importantly, the results of MTT assays and colony formation assays indicated that DDP treatments combined with lnc-MCEI knockdown significantly enhanced the chemosensitivity of KYSE-30 and EC109 cells to DDP (**Figure 3C and 3D**). In addition, the combination of these two strategies dramatically induced the apoptosis of KYSE-30 and EC109 cells (**Figure 3E**). All these data suggested that lnc-MCEI knockdown sensitizes ESCC cells to DDP.

### Lnc-MCEI functioned as a competing endogenous RNA (ceRNA) via sponging miR-6759-5p

**Figure 4:**
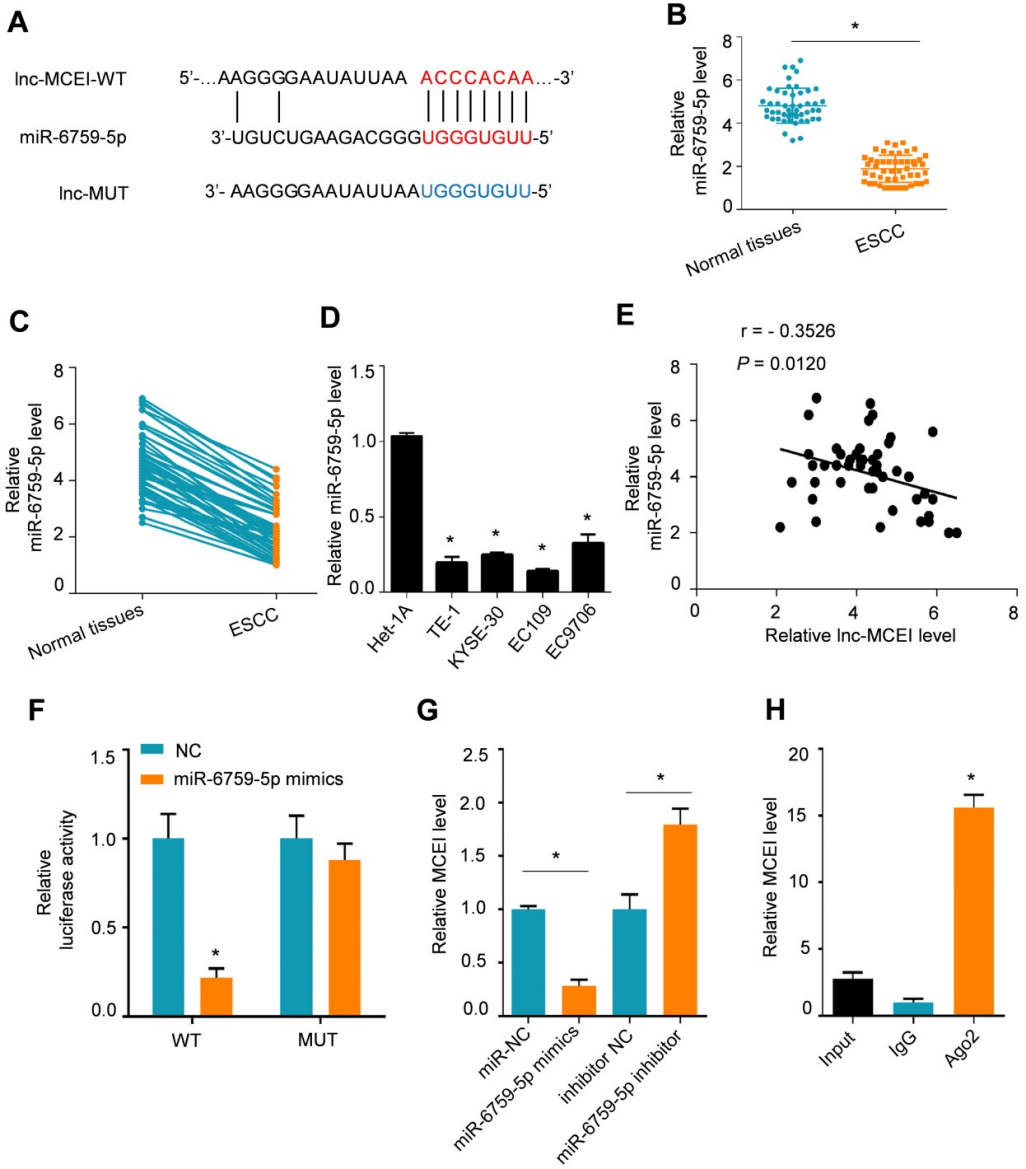
** Lnc-MCEI functioned as a competing endogenous RNA (ceRNA) via sponging miR-6759-5p. A.** The putative binding site of lnc-MCEI and miR-6759-5p was analysed by LncBase v2.0 database. **B.** The significantly down-regulated miR-6759-5p were observed in ESCC tissues compared to the normal controls. N = 50. **C.** The significantly down-regulated miR-6759-5p were also observed in ESCC tissues compared to the matched normal tissues. N = 50. **D.** miR-6759-5p was down-regulated in ESCC cell lines compared to the human esophageal epithelial cell line (Het-1A). **E.** A significant negative correlation between the expression of lnc-MCEI and that of miR-6759-5p. **F.** Luciferase activity of wild-type or mutated lnc-MCEI in HEK-293T cells examined by the dual luciferase reporter assays. **G.** Relative lnc-MCEI levels in KYSE-30 cells treated with miR-6759-5p mimics or miR-6759-5p inhibitor. **H.** RNA immunoprecipitation (RIP) were performed to examine the interaction between lnc-MCEI and miR-6759-5p. *P < 0.05.

To further explore the underlying mechanisms of lnc-MCEI involved in chemoresistance, the proposed target genes of lnc-MCEI were examined by searching in LncBase v2.0 database. The results indicated that lnc-MCEI could act as a ceRNA by binding to miR-6759-5p (**Figure 4A**). In addition, the decreased lnc-MCEI expression in ESCC tissues compared to the normal controls or matched normal tissues was observed (N = 50; **Figure 4B and 4C**) and lnc-MCEI expression was also decreased in ESCC cell lines compared to the human esophageal epithelial cell line was also observed (**Figure 4D**). Moreover, we found that there was a significant negative correlation between the expression of lnc-MCEI and that of miR-6759-5p (**Figure 4E**). Subsequently, the interaction between lnc-MCEI and miR-6759-5p was confirmed via dual luciferase reporter analysis and the data suggested that miR-6759-5p mimics decreased the luciferase activity of wild-type (WT) lnc-MCEI but had no effect on mutant (Mut) lnc-MCEI (**Figure 4F**). Additionally, the lnc-MCEI expression was affected by the miR-6759-5p levels in KYSE-30 cells treated with miR-6759-5p mimics or miR-6759-5p inhibitor (**Figure 4G**). Further RIP assays confirmed that Lnc-MCEI functioned as a ceRNA via sponging miR-6759-5p (**Figure 4H**).

### Lnc-MCEI mediated the chemosensitivity of ESCC by targeting miR-6759-5p/IGF2/PI3K/AKT axis

**Figure 5:**
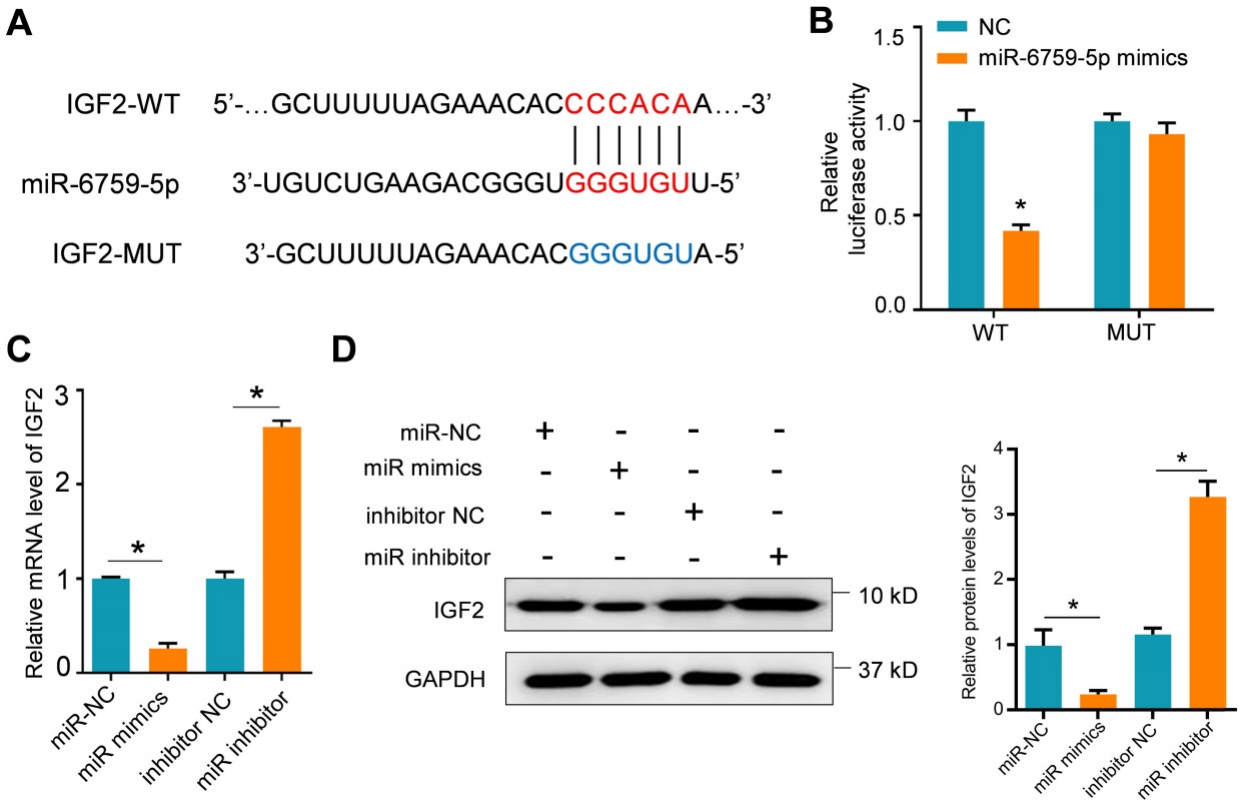
** IGF2 is a target of miR-6759-5p. A.** The putative binding site of lnc-MCEI and miR-6759-5p was analyzed by the TargetScan Human 7.2 database. **B.** Luciferase activity of wild-type or mutated 3'-UTR in HEK-293T cells determined by the dual luciferase reporter assays. **C.** The mRNA expression levels in KYSE-30 cells treated with miR-6759-5p mimics or miR-6759-5p inhibitor. **D.** The protein expression levels of IGF2 in KYSE-30 with the indicated treatments. *P < 0.05.

**Figure 6:**
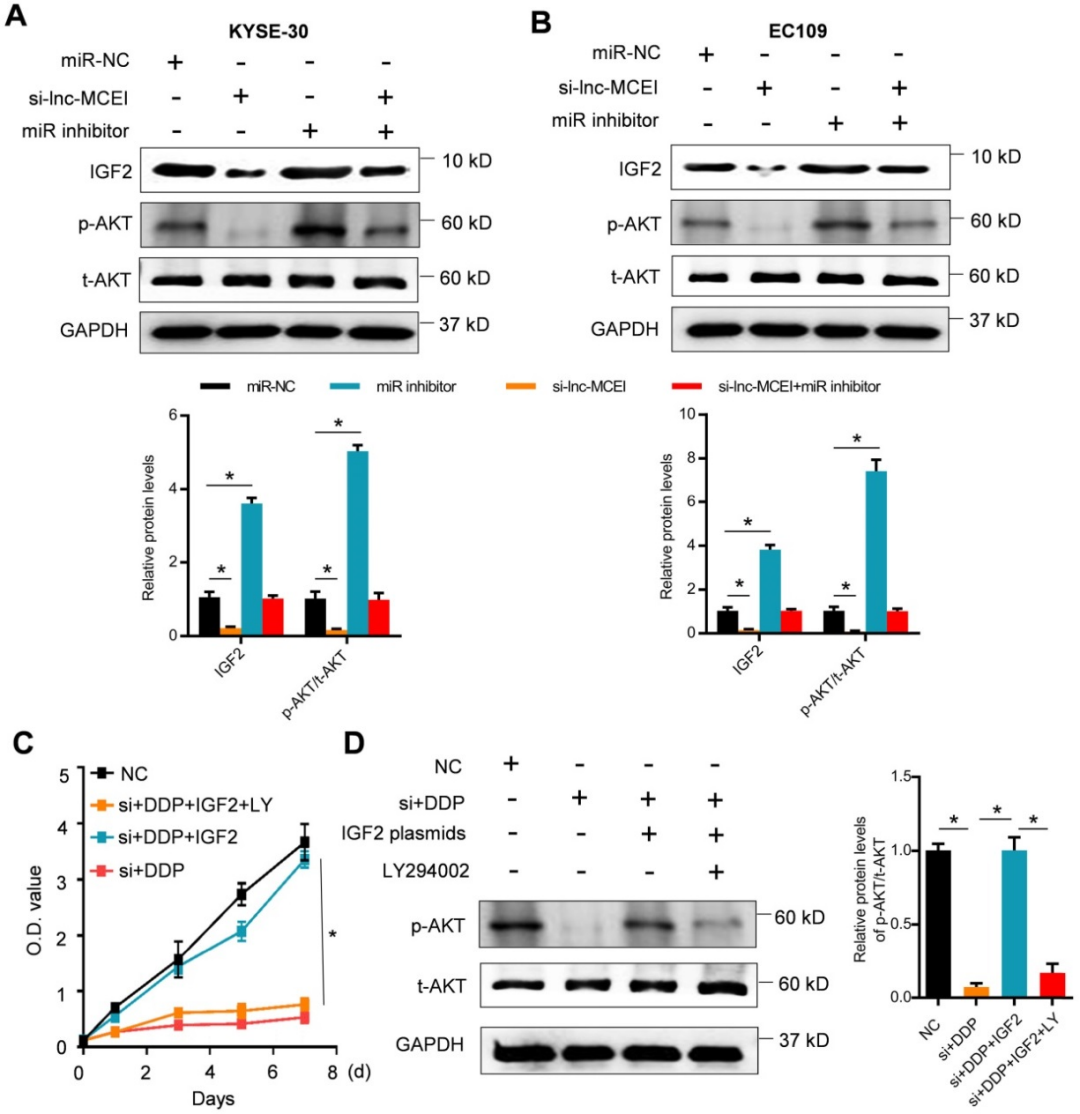
** Lnc-MCEI mediated the chemosensitivity of ESCC by targeting miR-6759-5p/IGF2/PI3K/AKT axis. A and B.** The protein expression levels of IGF2, phosphorylated AKT (p-AKT) and total AKT (t-AKT) in KYSE-30 and EC109 cells treated with si-lnc-MCEI alone or combined with miR-6759-5p inhibitor. **C.** MTT assays were performed to examine the cell proliferation in KYSE-30 cells treated with si-lnc-MCEI, DDP, IGF2 plasmids (IGF2) or the PI3K inhibitor LY294002 (LY). **D.** The protein expression levels of p-AKT and t-AKT in KYSE-30 with the indicated treatments. *P < 0.05.

The potential target genes of miR-6759-5p were further examined by using the TargetScan Human 7.2 database. The putative binding sites of miR-6759-5p were observed in 3'-UTR of IGF2 mRNA (**Figure 5A**). The results of dual luciferase reporter assays indicated that the luciferase activity of IGF2 with wild-type (WT) 3'-UTR was significantly decreased whereas IGF2 with mutant 3'-UTR was not influenced after miR-6759-5p mimics treatments (**Figure 5B**). The mRNA or protein IGF2 expression was also affected by the miR-6759-5p levels in KYSE-30 cells treated with miR-6759-5p mimics or miR-6759-5p inhibitor (**Figure 5C and 5D**). What's more, lnc-MCEI knockdown significantly decreased the protein expression IGF2 and phosphorylated AKT (p-AKT) but this effect was rescued by miR-6759-5p inhibitor in both KYSE-30 and EC109 cells (**Figure 6A and 6B**). In addition, MTT assays suggested that IGF2 overexpression rescued the inhibitory effect of the combination of lnc-MCEI knockdown and DDP whereas the effect of IGF2 overexpression was then abolished by the PI3K inhibitor LY294002 in KYSE-30 cells (**Figure 6C**). Further western blotting data exhibited consistent changes of IGF2 and p-AKT in KYSE-30 cells (**Figure 6D**). These data indicated that lnc-MCEI mediated the chemosensitivity of ESCC by targeting miR-6759-5p /IGF2/PI3K/AKT axis.

### The miR-6759-5p inhibitor or IGF2 overexpression restores the effect of lnc-MCEI knockdown on chemotherapy

**Figure 7:**
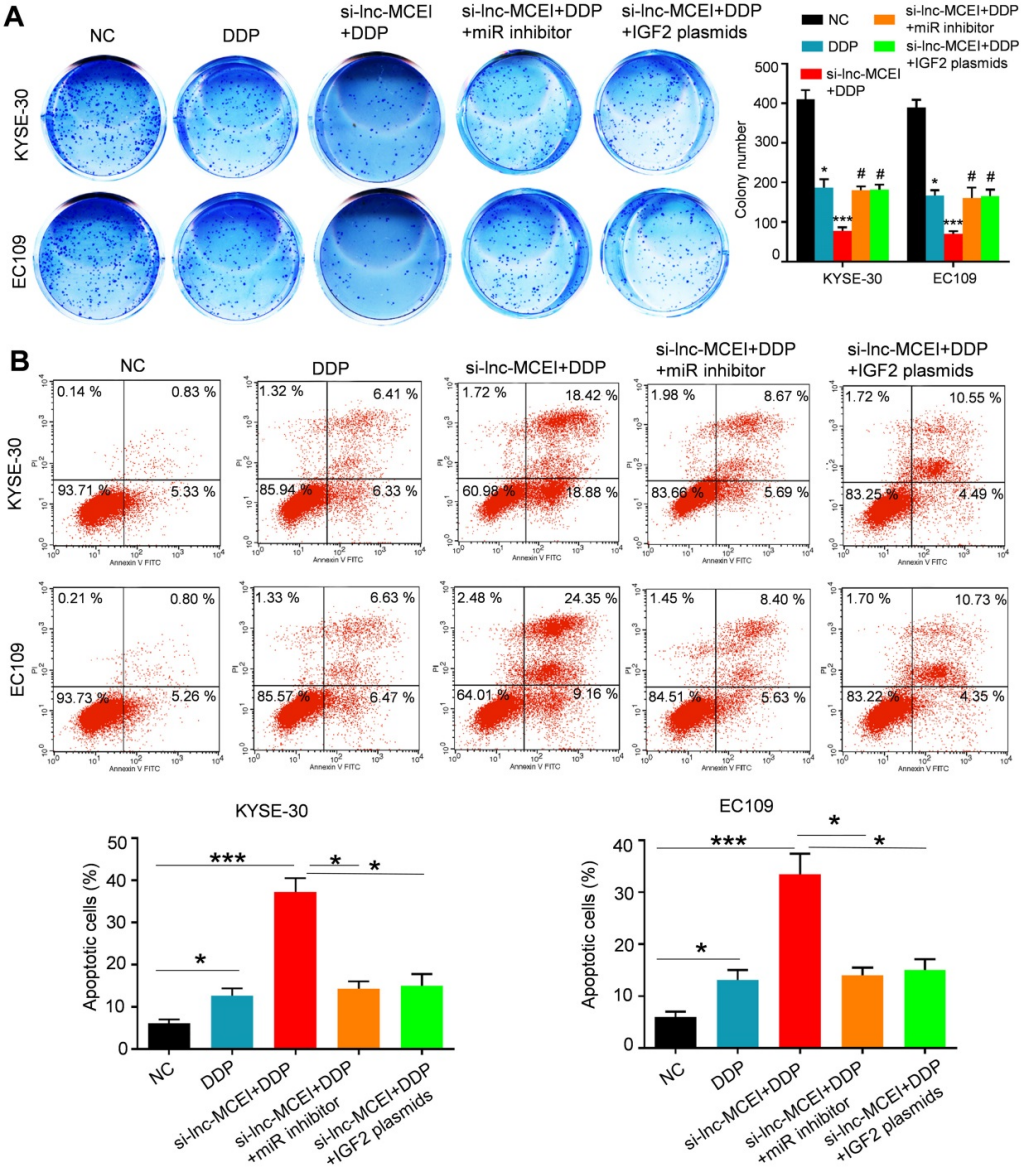
** The miR-6759-5p inhibitor or IGF2 overexpression restores the effect of lnc-MCEI knockdown on chemotherapy. A.** Colony formation assays in both KYSE-30 and EC109 cells with the indicated treatments (left) and quantification of colony numbers (right). **B.** Flow cytometry was performed to determine the percentage of apoptosis in various groups. *P < 0.05, **P < 0.01 and ***P < 0.001.

To further confirm the role of miR-6759-5p and IGF2 in the lnc-MCEI-mediated ESCC progression and chemoresistance, colony formation assays were performed and the data indicated that miR-6759-5p inhibitor or IGF2 overexpression rescued the inhibitory effect of the combination of lnc-MCEI knockdown and DDP (**Figure 7A**). Furthermore, the combination of lnc-MCEI knockdown and DDP significantly induced the apoptosis of KYSE-30 and EC109 cells but the effect was abolished by the miR-6759-5p inhibitor or IGF2 overexpression (**Figure 7B**). These data suggested that the miR-6759-5p inhibitor or IGF2 overexpression restores the effect of lnc-MCEI knockdown on chemotherapy.

### Lnc-MCEI inhibition enhances the ESCC chemotherapy to DDP in nude mice

**Figure 8:**
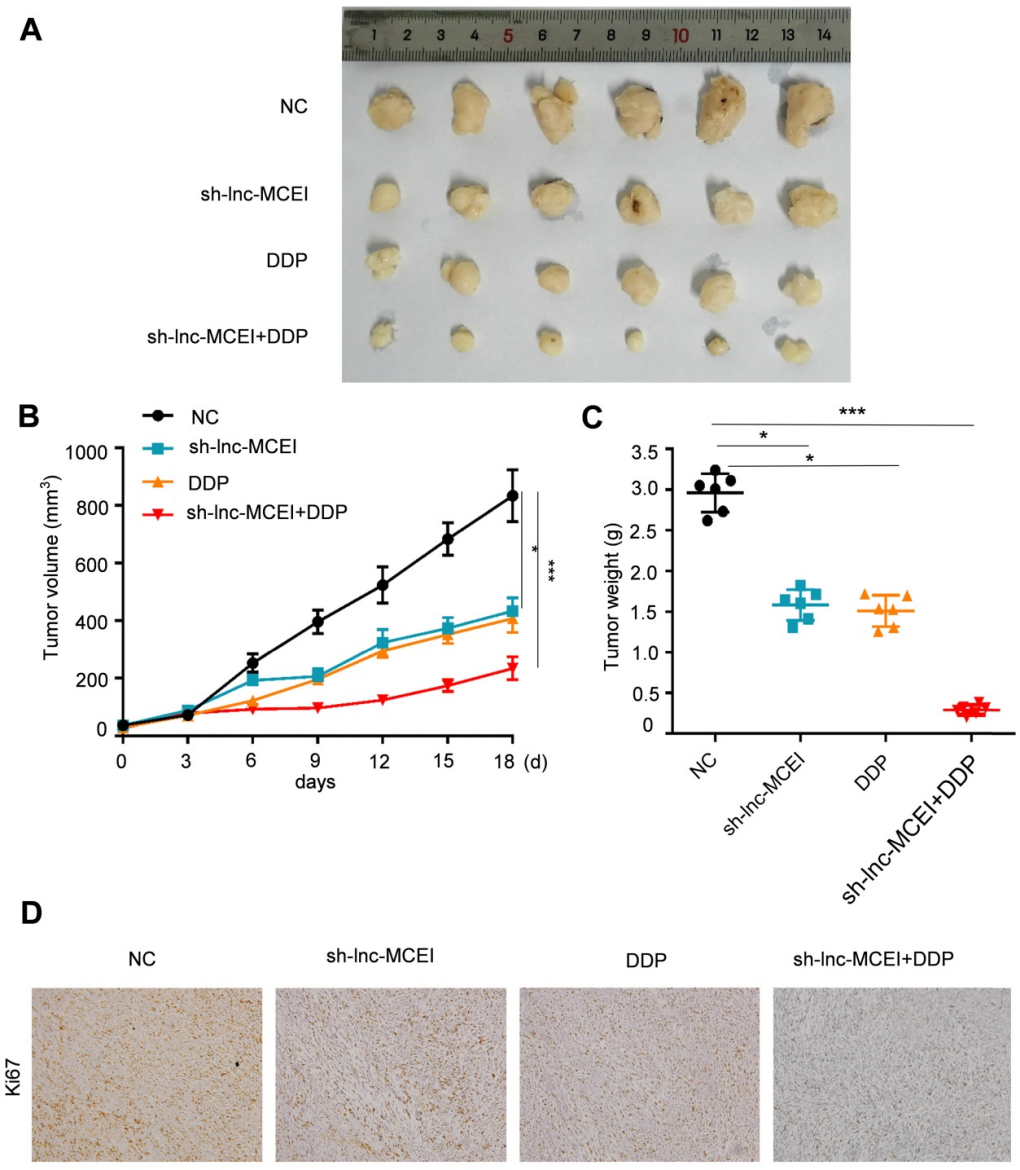
** Lnc-MCEI inhibition enhances the ESCC chemotherapy to DDP in nude mice. A.** Tumor images dissected from mice treated with sh-lnc-MCEI and DDP alone or combined the both.** B.** Tumor volume in the indicated groups. **C.** Tumor weight in the indicated groups. **D.** Representative images of Ki67 staining in the indicated groups. N = 6; *P < 0.05, **P < 0.01 and ***P < 0.001.

We next examined the role of lnc-MCEI in ESCC tumorigenesis* in vivo*. Immunodeficient nude mice were injected with EC109 cells that had been stably transfected with lnc-MCEI-shRNA (sh-MCEI) or negative control (NC). The results disclosed that tumor volume and tumor weight were significantly decreased in the sh-MCEI and DDP groups and the combination of sh-MCEI and DDP exhibited a more significant suppressive effect on the tumorigenesis (**Figure 8A-C**). Moreover, the positive rate of Ki67 was also lower in the sh-MCEI + DDP group than the sh-MCEI or DDP group (**Figure 8D**). In summary, our data suggested that lnc-MCEI inhibition suppressed ESCC tumorigenesis *in vivo* and lnc-MCEI mediated the chemosensitivity of ESCC by targeting miR-6759-5p /IGF2/PI3K/AKT axis.

## Discussion

The mortality of esophageal squamous cell carcinoma (ESCC) remains high despite the rapid promotion in diagnosis and treatment of ESCC 25, 26. ESCC tumorigenesis has been proved to be regulated by complex mechanisms including cytoplasmic enzymes 27, various oncogenes 28, receptor tyrosine kinases 29, tumor suppressor genes 30 and lncRNAs 14. In the present study, we found that lnc-MCEI promotes ESCC tumorigenesis and chemoresistance. Lnc-MCEI knockdown significantly inhibits the malignant biological behaviors of ESCC cells and sensitizes ESCC cells to DDP. Further mechanism disclosed that lnc-MCEI functioned as a competing endogenous RNA (ceRNA) via sponging miR-6759-5p and lnc-MCEI mediated the chemosensitivity of ESCC by targeting miR-6759-5p/IGF2/PI3K/AKT axis. Our results suggested that lnc-MCEI was a promising therapeutic target for ESCC.

Lnc-MCEI has been rarely studied in previous studies and its role in cancer has never been discovered. Shi-Mei Zhuang et al. reported that lnc-MCEI was one of the candidate lncRNAs frequently amplified in hepatocellular carcinoma (HCC) but the function in HCC progression have been uncovered 20. In addition, bioinformatics analysis demonstrated that lnc-MCEI might participate in ESCC tumorigenesis and chemosensitivity based on the published microarray data (GSE89102) which tends to analyze the differential expressed genes (DEGs) between the 5 paired ESCC tissues and normal tissues 17. Thus, we focused on the role of lnc-MCEI in ESCC and aimed to discover the biological function. Further *in-vivo* and *in-vitro* biological experiments in our study confirm that lnc-MCEI promotes the malignant biological properties and inhibition of lnc-MCEI sensitizes ESCC cells to DDP. Therefore, lnc-MCEI was identified as an oncogene for the first time in our study.

Studies have shown that lncRNAs may act as a competing endogenous RNA (ceRNA) sponging for miRNAs via the miRNA response elements (MREs) 31-33. The present results in this study indicated that lnc-MCEI could function as a ceRNA by sponging miR-6759-5p. A global microRNA profiling of metastatic conjunctival melanoma demonstrated that miR-6759-5p was upregulated in metastatic primary conjunctival melanoma 34. However, miR-6759-5p was proved to be downregulated in ESCC tissues and it is involved in the ESCC tumorigenesis. However, the axis of lnc-MCEI-miR-6759-5p was just one of the mechanisms involved in the ESCC progression. For example, bioinformatics analysis also demonstrated that lnc-MCEI may sponge miR-6793-5p. Thus, we will put more effort into the molecular mechanism exploration in our future studies.

IGF2 was increased 1.97-fold in esophageal cancer than that in normal tissues detected by immunohistochemistry (IHC) 35. Previous studies pointed out that lncRNA 91H had a positive effect on the ESCC by the inhibition of IGF2 36. Additionally, IGF2 promotes the stemness of ESCC cells by inducing CD133 expression 37. Moreover, the epigenetically changes of IGF2 was associated with prognosis of ESCC 38. Thus, IGF2 promote ESCC tumorigenesis and our data confirm this. In our study, IGF2 was identified as a target of miR-6759-5p. What's more, studies have revealed that miR-145 sensitizes ESCC to cisplatin via direct inhibition of PI3K/AKT signaling. Therefore, lnc-MCEI/miR-6759-5p/IGF2/PI3K/AKT axis was clarified to be involved in the lnc-MCEI-mediated chemoresistance of ESCC in the present study 24.

Taken together, the beneficial effect of lnc-MCEI knockdown on the malignant biological behaviors of ESCC cells and chemosensitivity of ESCC cells to DDP was verified in the present study. Therefore, lnc-MCEI was identified as an oncogenic lncRNA in ESCC and could be a potential therapeutic target for ESCC by inhibition of miR-6759-5p/IGF2/PI3K/AKT axis.

## Abbreviations

ceRNA: competing endogenous RNA
DDP: cisplatin
DEGs: differential expressed genes
EAC: esophageal adenocarcinoma
EZH2: enhancer of zeste homolog 2
ESCC: esophageal squamous cell carcinoma
5-FU: 5-Fluorouracil
GEO: Gene Expression Omnibus
HCC: hepatocellular carcinoma
IGF2: insulin-like growth factor-2
IHC: immunohistochemistry
lncRNAs: long non-coding RNAs
MREs: miRNA response elements
NC: negative control
non-pCR: non-pathological complete response
pCR: pathological complete response
qPCR: quantitative real-time polymerase chain reaction
RIP: RNA immunoprecipitation
shRNA: hairpin RNA
siRNA): small interfering RNA
